# Ecosystem Services Flows: Why Stakeholders’ Power Relationships Matter

**DOI:** 10.1371/journal.pone.0132232

**Published:** 2015-07-22

**Authors:** María R. Felipe-Lucia, Berta Martín-López, Sandra Lavorel, Luis Berraquero-Díaz, Javier Escalera-Reyes, Francisco A. Comín

**Affiliations:** 1 Instituto Pirenaico de Ecología-CSIC, Av. Nuestra Señora de la Victoria, s/n, 22700 Jaca, Huesca, Spain; 2 Social-Ecological Systems Laboratory, Department of Ecology, Edificio de Biología, Universidad Autónoma de Madrid, C/ Darwin 2, 28049, Madrid, Spain; 3 Environmental Change Institute, Oxford University Centre for the Environment, South Parks Road, Oxford, OX1 3QY, United Kingdom; 4 Laboratoire d’Ecologie Alpine, CNRS, Université Grenoble Alpes, BP 53, 38041, Grenoble Cedex 9, France; 5 Social and Participatory Action Research Group (GISAP), Department of Social Anthropology, Basic Psychology, and Public Health, Universidad Pablo de Olavide, Carretera Utrera, km. 1, 41013, Sevilla, Spain; 6 Instituto Pirenaico de Ecología-CSIC, Av. Montañana 1005, 50192, Zaragoza, Spain; University of Lleida, SPAIN

## Abstract

The ecosystem services framework has enabled the broader public to acknowledge the benefits nature provides to different stakeholders. However, not all stakeholders benefit equally from these services. Rather, power relationships are a key factor influencing the access of individuals or groups to ecosystem services. In this paper, we propose an adaptation of the “cascade” framework for ecosystem services to integrate the analysis of ecological interactions among ecosystem services and stakeholders’ interactions, reflecting power relationships that mediate ecosystem services flows. We illustrate its application using the floodplain of the River Piedra (Spain) as a case study. First, we used structural equation modelling (SEM) to model the dependence relationships among ecosystem services. Second, we performed semi-structured interviews to identify formal power relationships among stakeholders. Third, we depicted ecosystem services according to stakeholders’ ability to use, manage or impair ecosystem services in order to expose how power relationships mediate access to ecosystem services. Our results revealed that the strongest power was held by those stakeholders who managed (although did not use) those keystone ecosystem properties and services that determine the provision of other services (i.e., intermediate regulating and final services). In contrast, non-empowered stakeholders were only able to access the remaining non-excludable and non-rival ecosystem services (i.e., some of the cultural services, freshwater supply, water quality, and biological control). In addition, land stewardship, access rights, and governance appeared as critical factors determining the status of ecosystem services. Finally, we stress the need to analyse the role of stakeholders and their relationships to foster equal access to ecosystem services.

## Introduction

The ecosystem services framework [1] has enabled the broader public to acknowledge the benefits nature provides to humans [2]. These include tangible or material benefits such as provisioning services (e.g., food, raw materials) and intangible or immaterial benefits such as cultural services (e.g., recreation, relaxation, environmental education, and aesthetic enjoyment), regulating services (e.g., nutrient regulation and climate regulation), and supporting ecosystem properties (i.e., the underlying mechanisms of the ecosystems) such as habitat provision and soil formation. However, not everybody benefits equally from these ecosystem services. Recent research highlighted spatial characteristics as drivers of inequalities in ecosystem services provision [3,4]. For example, whereas ‘upstream’ populations may benefit from water quality, ‘downstream’ populations may not. Yet, the potential of ecosystems to benefit humans not only depends on the spatial characteristics of the flow of services [5–8] but are derived from their multiple types of interactions [9,10]. On the one hand, these depend on the interactions among ecosystem properties and ecosystem services causing trade-offs and synergies [11,12]. On the other hand, the interactions among stakeholders, which are partially caused by power relationships, can determine the access to and management of ecosystem services.

Power relationships are a well-known concept used in natural resource management to determine asymmetries in the access to resources [13–18]. Power relationships are also well-known in social sciences and are used to uncover the inherent asymmetries in social relations [19–23]. For instance, ecological anthropology and political ecology incorporate the concept of power to human-environment interactions [24]. In ecosystem services literature, studies analysing power relationships are developed in the context of payments for ecosystem services [25,26]. In this paper we investigate how power relationships modulate either the stakeholders’ use of ecosystem services or the interactions between the ecosystem services supplied. Power asymmetries among stakeholders mean that some stakeholders may use a particular ecosystem service or a set of ecosystem services while other stakeholders might be excluded. Therefore, power asymmetries can create social conflict [4,27], and affect stakeholders’ well-being [28]. For instance, empowered stakeholders can decide about the ecosystem services supplied and regulate access to them, negatively affecting non-empowered stakeholders by reducing their ability to access ecosystem services. In addition, management decisions ultimately driven by power relationships modulate ecosystem services interactions resulting in trade-offs between ecosystem services [9,29]. Therefore, power relationships emerge as a key factor influencing: (i) stakeholders’ access to ecosystem services; (ii) stakeholders’ interactions and roles regarding ecosystem services; and (iii) environmental management shaping the provision of ecosystem services.

Including the concept of power relationships into ecosystem services research exposes the gap between the production of services by an ecosystem and the actual benefits stakeholders receive. Such gaps can reveal those stakeholders dependent on certain ecosystem services for their well-being that are at risk of being excluded from accessing ecosystem services [28]. Power relationships, including the beneficiaries of ecosystem services, the contributors to services production, and those who are excluded (i.e., the losers [30]) have not yet been integrated into ecosystem services management [31]. Integrating power relationships into ecosystem services research explicitly provides an opportunity to assess how power mediates ecosystem services flows that may be crucial information to design more sustainable management policies.

In this context, the aim of this study was to reveal the role of power relationships for ecosystem services flows from the supply by the ecosystems to the users. In order to address this general aim, in the next section we describe the adaptation of the ecosystem services ‘cascade’ framework to integrate the analysis of ecological interactions among ecosystem services and of power asymmetries among stakeholders that determine the use and management of ecosystem services. Then we describe the methods used to apply the conceptual framework to the River Piedra case study in northeastern Spain. The results section shows the main findings related to the dependence relationships among the ecosystem services analysed and the role of stakeholders mediating access to ecosystem services through the identification of power asymmetries. In the discussion section we address the applicability of the conceptual framework and the implications for accessing ecosystem services of both power imbalances among stakeholders and the excludable and rival characteristics of ecosystem services. Finally, we provide some insights for environmental management to deal with social-ecological interactions along the flow of ecosystem services.

## Conceptual Framework

**Fig 1:**
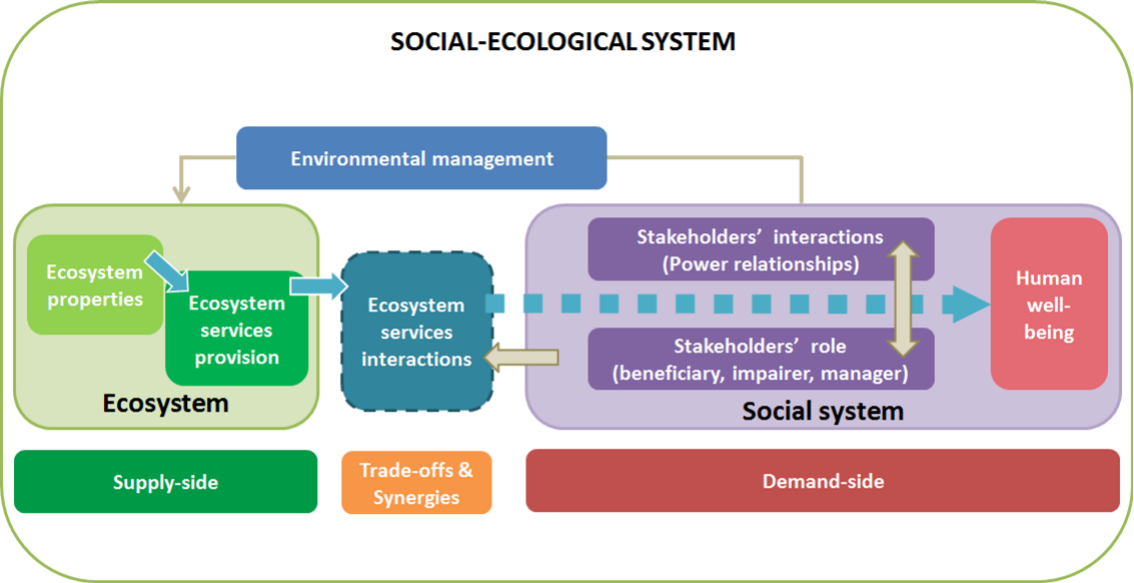
Conceptual framework of the interactions along the flow of ecosystem services from the supply-side to the demand-side and human well-being. Blue arrows represent the flow of ecosystem services. Beige arrows denote interactions within or from the social system (Inspired from Haines-Young and Potschin [32], Martín-López et al. [34], Spangenberg et al. [36]).

The ‘cascade’ framework depicts ecosystem services as a flow from the ecosystem towards human well-being [32]. This framework has been gradually modified to incorporate ongoing developments of ecosystem service science [33–35], such as the introduction of societal processes in the step from ‘service’ to ‘benefit’ [36]. We propose to further refine this step by identifying both the interactions among ecosystem services and among stakeholders that mediate and could impair stakeholders’ access to ecosystem services (Fig 1).

Ecosystem properties (i.e., the biophysical structure and functioning of ecosystems) contribute to provide ecosystem services and human well-being. However, ecosystem services are not isolated independent units, but rather depend on each other [37] and interact causing trade-offs and synergies [12] (see Table 1 for definitions). Some of these interactions can be determined by the use and management of ecosystem services performed by stakeholders [9,29,38]. Thus, the flow of ecosystem services is shaped through the social system (i.e., stakeholders’ interactions, roles, and preferences) by several types of complex interactions among multiple stakeholders. First, stakeholders interact among themselves through different types of relationships that are modulated by formal power asymmetries (e.g., property rights, access, or legal permissions), informal power asymmetries (e.g., social leadership, gender inequity), or hidden power imbalances (e.g., social pressure promoting self-censorship). Second, stakeholders play different roles in the management and use of ecosystem services. We identify two main roles for stakeholders: they can manage ecosystem services (i.e., co-producing or impairing them), or be recipients of ecosystem services (i.e., using them but also being excluded from access) [31]. A single stakeholder could perform several of these roles [39]. In addition, stakeholders’ interactions affect the role of individual stakeholders in the system, which in turn perpetuates their power relationships [40–44]. The social system drives environmental management, establishing the management and use of ecosystem services and conditioning the ecosystem properties responsible for ecosystem service provision [45,46].

**Table 1:** Key concepts related to ecosystem services and definitions. Concepts are listed according to the order they appear in the text.

Concept	Definition
Trade-off	Situation in which land use or management actions increase the provision of one ecosystem service and decrease the provision of another. This may be caused by simultaneous responses to the same driver or caused by true interactions among ecosystem services (adapted from [12]).
Synergy	A win-win situation that involves a mutual improvement of two ecosystem services (adapted from [38]).
Stakeholder	Any group, organization or individual having a stake, interest, or who can affect a biological or physical resource, ecosystem service, institution or social system, or someone who is or may be affected by a public policy (adapted from [29,38]).
Power relationships	The human ability to control or influence the access of others to ecosystem services.
Beneficiary	Stakeholders who directly use and benefit from ecosystem services [39].
Impairer	Stakeholders who negatively affect the provision of ecosystem services as a consequence of their direct or indirect use (adapted from [39]).
Manager	Stakeholders who directly influences the way ecosystem services are provided or can be used [39].

## Methods

### Study area

**Fig 2:**
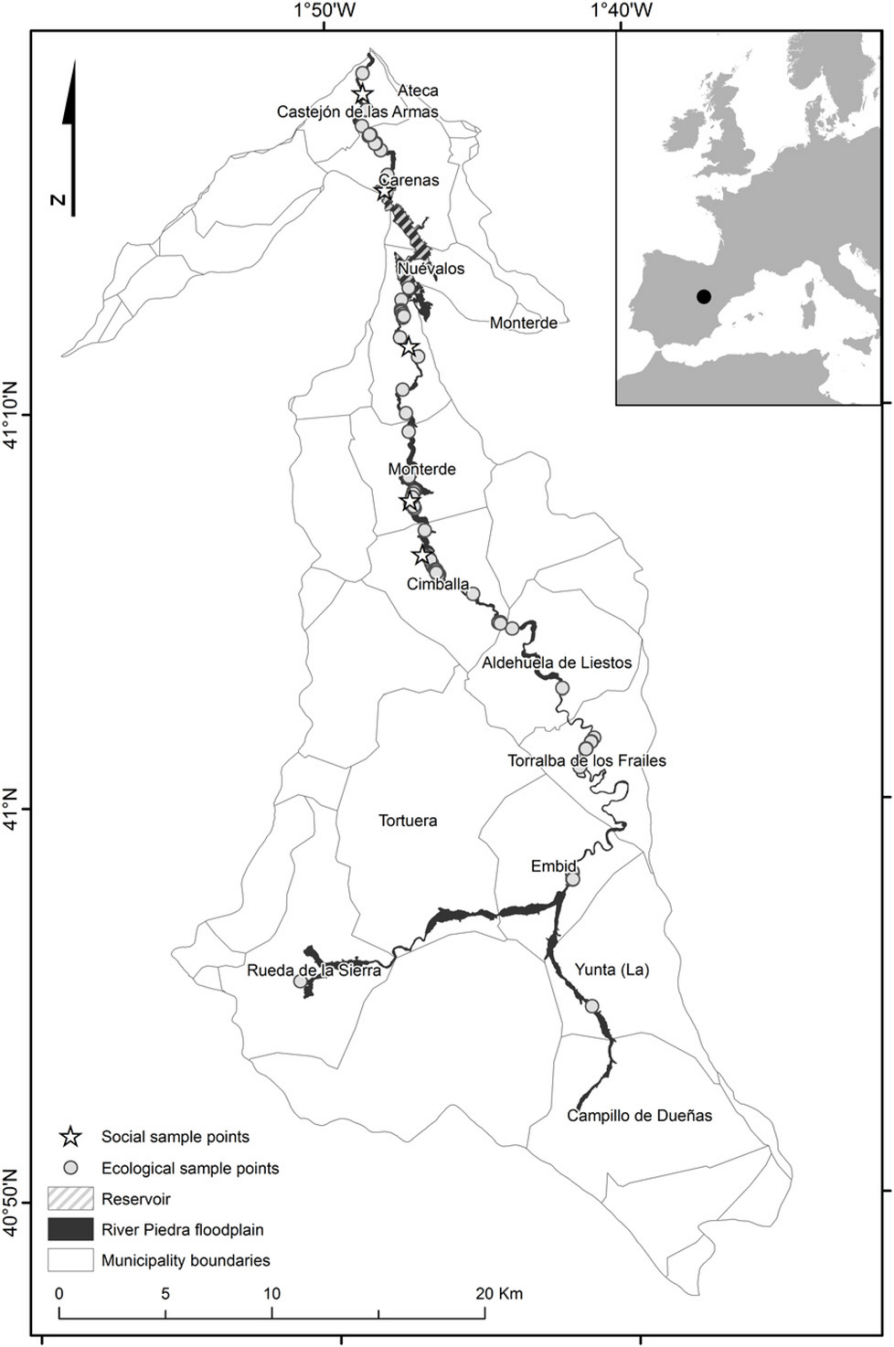
The watershed of the River Piedra in NE Spain divided by municipality boundaries. Dots indicate ecological sample points and stars social sample points (note that external stakeholders are not represented in this figure).

The study area comprises the municipalities across the River Piedra (616 km^2^) in north-eastern Spain (Fig 2). The region is characterized by marked seasonal variability in the water flow. The upper part of the River Piedra (ca. 46 km) is dry for most of the year due to a combination of a semiarid climate and a calcareous substrate. The middle part of the river is permanent (ca. 30 km), as it receives groundwater discharge. River flow rates in the lower lands are usually altered with respect to natural rates depending on La Tranquera reservoir operations. This 78800000 m^3^ reservoir is regulated primarily for the benefit of irrigators from others basins.

The land cover of the floodplain is characterized by agricultural use (76%), including dryland cereal crops in the upper lands, irrigated cereal crops and poplar groves in the middle part, and fruit groves and orchards in the lower lands. Riparian forests (4.2%) are restricted to the upland gorges (usually dry) located between the municipalities of Aldehuela de Liestos and Embid, and to a private natural park, the Monasterio de Piedra, located in the municipality of Nuévalos. The park’s main attraction is the large number of waterfalls of the River Piedra, which contrast hugely with the semiarid surrounding landscape. The tourism generated by the park is the main economic driver of the area, and attracts tourists to other nearby amenities and activities (e.g., restaurants, lodges, trekking, mountain-biking, ornithology, fishing, kayaking). The population is dominated by elderly people and significantly more men than women, although this trend reverses during school holidays.

### Data collection

#### Ecosystem services supply

We identified the key ecosystem services provided by floodplains following Harrison et al. [47] at European scale, and Vidal-Abarca and Suárez [48] at national scale, as well as from prior knowledge of the functioning and the ecosystem services of the study area [29]. We gathered available data of 12 ecosystem services that were relevant to maintain the flow of services in the area. The selection included two supporting ecosystem properties (soil conditions, composed of soil formation and soil stability, and habitat quality), four regulating services (nutrient regulation, carbon sequestration, biological control, and water quality), three provisioning services (freshwater supply, food production, and raw materials), and three cultural services (aesthetic, recreation, and environmental education). Table 2 synthesizes the methods and indicators used for measuring each ecosystem service. For further details about methods see S1 File.

**Table 2:** Description of the supporting ecosystem properties and ecosystem services identified as relevant to maintain the flow of services in the River Piedra floodplain.

Category	Type	Name	Indicator	Method (units)	Number of sample points	Years collecting data	Reference
Supporting	Intermediate	Habitat quality	RQI	Riparian Quality Index (unitless)	21	2011, 2012	[49]
Supporting	Intermediate	Soil conditions	Soil formation	Organic matter content in top soil (percentage)	324	2011, 2012	[50]
			Soil stability	Organic matter layer in top soil (cm)	324	2011, 2012	[51]
Regulating	Intermediate	Water quality	NO_2_^-^	Nitrite content in water (ppm)	281	2009–2011	[52]
			NO_3_^-^	Nitrate content in water (ppm)	281	2009–2011	[52]
			PO_4_^-^	Phosphate content in water (ppm)	281	2009–2011	[52]
Regulating	Intermediate	Nutrient regulation	C	Total carbon in top soil (percentage)	324	2011, 2012	[53]
			N	Total nitrogen in top soil (percentage)	324	2011, 2012	[29]
			P	Total phosphorus in top soil (percentage)	324	2011, 2012	[54]
Regulating	Intermediate	Biological control	Vertical vegetation structure	Number of different vertical vegetation structures (number)	54	2011–2013	[55]
Regulating	Final	Carbon sequestration	CO_2_ sequestration	CO_2_ equivalent tons sequestered by plants (CO_2_ eq tons/year)	21	2011	[56]
Provisioning	Final	Freshwater supply	Water consumption	Cubic meters of water concessions per municipality (m^3^/year)	12	2011	[57]
Provisioning	Final	Food production	Yield	Kilograms per hectare (kg/Ha · year)	21	2011	[58]
			Calories	Kilocalories per hectare (kcal/Ha · year)	21	2011	[58]
			Gross value	Euros per hectare (€/Ha · year)	21	2011	[59]
Provisioning	Final	Raw materials	Production	Tons of annual biomass increase (tons/year)	21	2011	[56]
Cultural	Final	Aesthetic	Pictures	Number of pictures uploaded to Panoramio (number)	84	2014	[60]
Cultural	Final	Recreation	Fishing	Meters of river available for fishing (m)	84	2012	[61]
			Sports	Extent of floodplain viewshed from open access trails (Ha)	84	2012	[62]
			Picnic areas	Number of designed picnic areas (number)	84	2012	[29]
Cultural	Final	Environmental education	Information panels	Number of panels with information about the ecosystem (number)	84	2012	[29]

#### Ecosystem services benefits

To learn about the ecosystem services used in the floodplain of the River Piedra and the limitations to benefiting from these services, we conducted 71 face-to-face, semi-structured interviews with the main stakeholders of the study area. These included residents, holidaymakers, farmers, tour operators (hosting or guiding nature tourists), local mayors, local teachers, scientists, nature protection agents, and technicians working on riverbank restoration projects. The targeted local population comprised 880 inhabitants [63] from five municipalities (see Fig 2). The municipalities within the seasonal river flow were excluded from this study as they do not perceive themselves to live within a riparian ecosystem and their activities depend little on this ecosystem.

Table 3 presents a classification of stakeholders and a brief description. Interviewees were asked about the status of the riparian ecosystem, the causes and solutions to solve the problems identified, and about the uses, products, and benefits they derived from the valley of the River Piedra. A minimum number of ten stakeholders from each of the main stakeholders’ groups were interviewed until the information received was saturated (i.e., we did not receive any new information from the same sector of stakeholders [64]). Interviews were performed by the first author between August 2011 and March 2012 and lasted between 30 and 90 minutes. Digital records of the interviews were made with the interviewees’ consent. Interviews were transcribed and coded for further analysis (see Table A in S2 File for details of the interviewees).

**Table 3:** Stakeholders’ groups, number of respondents, and description.

Group	Name	n	Description
1	Primary sector	16	Farmers (including both land owners and land tenants of orchards, fruit groves, irrigated and dry cereal crops, and poplar groves), shepherds, and workers at a fish farm.
2	Recreation sector	13	Owners or workers at restaurants, hotels, lodges, nature tour operators, adventure enterprises, and at the Monasterio de Piedra (i.e., a regional touristic site).
3	Leisure	26	Retired residents, visitors, hikers, bikers, fishermen, etc.
4	Institutions	16	Local councils.
			Government bodies: the regional water management body (Confederación Hidrográfica del Ebro), which depends on the Ministry of the Environment; Nature Protection Agents, which depend on the regional government (Gobierno de Aragón).
			Scientific and educational institutions: scientists from the Pyrenean Institute of Ecology (IPE – CSIC) and the University of Zaragoza; teachers from the local elementary school and high school.
			Technicians from a public company working on environmental projects on the riverbanks and the floodplain of the River Piedra.

### Data analysis

#### Ecosystem services supply

**Fig 3:**
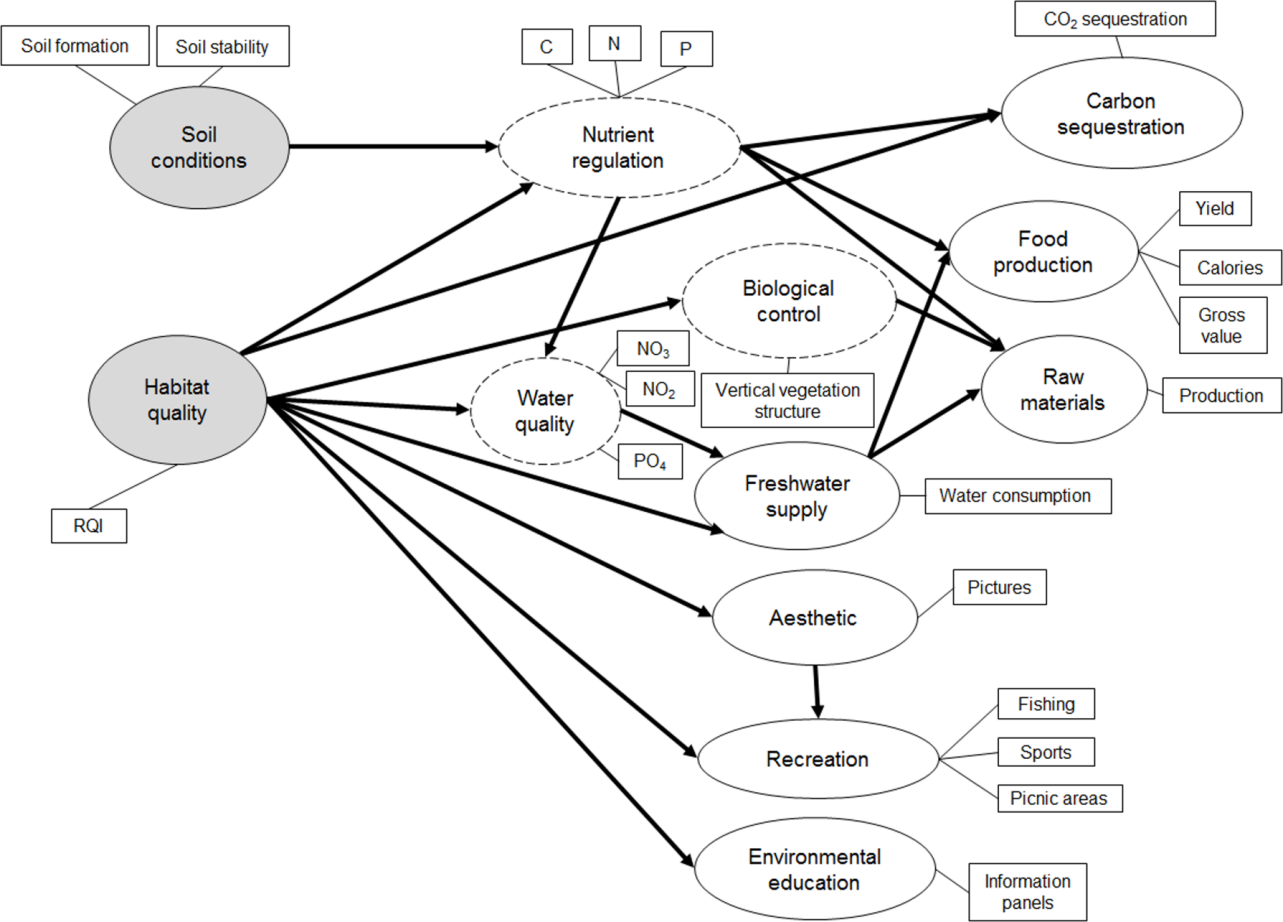
Conceptual diagram of the initial structural equation model (SEM) based on those paths among variables determined by the expert panel. Latent variables (i.e., ecosystem properties or services) are denoted by ellipses, while manifest variables (i.e., the indicators used) are inside a box. Supporting ecosystem properties (i.e., exogenous latent variables) are shaded, intermediate regulating services are dashed, and final services are solid.

To model the flow of ecosystem services, we built an initial path model (Fig 3) on the basis of the classification of ecosystem services as intermediate or final. To build the path model, we convened an expert panel in May 2014 composed of four experts from the fields of ecosystem service science, conservation ecology, and limnology. The experts independently modelled the flow of ecosystem services in the study area. To assess the performance of the model we used structural equation modelling (SEM), a statistical technique to model complex multivariable relationships. SEM includes two models: the relations among all latent variables (i.e. the ecosystem services), and the relations between the manifest (observed) variables and their own latent variable (i.e. between the indicators used (Table 2) and the ecosystem services estimated) [65].

In our model, supporting ecosystem properties were considered as exogenous latent variables (i.e., independent on other ecosystem services), and intermediate regulating services and final services as endogenous latent variables (i.e., dependent on other ecosystem services) (Fig 3). Thus, in the River Piedra case study, soil conditions and habitat quality were the supporting ecosystem properties from which ecosystem services depended directly (linked by an arrow) or indirectly (linked through an intermediate regulating service). Nutrient regulation is a regulating service which directly depended on soil conditions and habitat quality. Carbon sequestration depended on habitat quality (because the quantity of trees mediates carbon sequestration) and nutrient regulation (also mediates trees’ performance). Biological control depended on habitat quality as the number of vertical vegetation structures hosting species performing different functions in the ecosystem depends on it. Water quality was related to habitat quality (e.g., a good quality of riparian habitats avoids runoffs into water) and to nutrient regulation (e.g., through regulating nitrogen and phosphorus content in soils). Freshwater supply was connected to both habitat and water quality as water supplied needs to be in a good ecological and chemical status which is mediated by a good habitat quality. Food and raw materials production was related to freshwater supply (e.g., increasing water for irrigation increases productivity) and to regulating services (nutrient regulation and biological control), whereas cultural services were directly linked to habitat quality.

We followed a formative SEM approach [66], in which each latent variable is related to its manifest variable by a linear function plus a residual term. We normalized all manifest variables to ensure homogeneous weights and checked the unidimensionality of the blocks of manifest variables using two criteria: i) Cronbach’s alpha greater or equal to 0.7 [67] and ii) Dillon-Goldstein’s rho greater or equal to 0.7 [68]. Manifest variables not meeting these criteria were dropped from the initial model. The quality of the final model was assessed using: i) the Relative Goodness of Fit index [69]; ii) the adjusted R^2^ of the latent variables; and iii) the average communalities [70]. All statistical analyses were performed with the software XLSTAT (2014.3.01).

#### Ecosystem services benefits

The ecosystem services mentioned by each stakeholder group during the interviews (see Table B in S2 File) provided evidence of their role in relation to ecosystem services. According to this information, we linked each stakeholder group to the services they used, contributed to produce, or impaired. Additionally, we classified the ecosystem services identified within a gradient from rival to non-rival, and from excludable to non-excludable through an expert panel. The panel was held in June 2014 and comprised six experts from the fields of ecosystem services, policy, and land management. Each of the experts independently classified ecosystem services according to the characteristics of this case study. In cases of divergence, the moderator of the panel unified the classifications according to the comments provided by each expert. We followed the approach of Costanza [71] (p. 351) which defined rival ecosystem services as those that can be consumed (“*the degree that one person’s benefiting from them interferes with or is rival with other’s benefiting from them”*), and excludable ecosystem services as those that can be privatized (“*the degree that individuals can be excluded from benefiting from them”*), and we incorporated the concept of congestible (i.e., moving from non-rival to rival if excessive use decreases their good initial conditions) suggested by Fisher et al. [3]. We used this classification to represent each ecosystem service in a diagram showing stakeholders’ use versus ability to manage ecosystem services by adapting the approach proposed by Reed et al. [17] and Iniesta-Arandia et al. [72], where the former identified four clusters according to the degree of power and interest of stakeholders and the latter according to their degree of dependence and influence. Additionally, we included a variant of this diagram displaying stakeholders’ use versus their ability to impair ecosystem services.

#### Ethics statement

Part of these analyses is based on interviews results. The interviewees were voluntary, and their answers were confidential and anonymized for analysis. Participants verbally consented to participate in this study under these conditions. Written consent was not requested in order to facilitate the interactions between interviewer and participants. We cannot document participant consent because we only started recording once participants had given their agreement. When participants did not agree to be recorded but consented to participate, written notes were taken by the interviewer. The Academic Commission of the Doctorate Program in Environment and Society of the Universidad Pablo de Olavide (Seville, Spain) approved this study and this consent procedure. Additionally, the Instituto Pirenaico de Ecología – CSIC, approved the methods used in field sampling.

## Results

### Dependence relationships among ecosystem services on the supply side

The SEM model explained a large proportion of the variation in the flow of ecosystem services, with a Relative Goodness of Fit of 0.858 (Table 4). A greater part of the variance was explained for environmental education (R^2^_a_ = 0.817), recreation (R^2^_a_ = 0.784), and nutrient regulation (R^2^_a_ = 0.488). Less variance (R^2^_a_ < 0.45) was explained for carbon sequestration, biological control, and raw materials.

**Table 4:** Latent variables, adjusted R^2^ (R^2^_a_), average communality (Ave. Com.), and Dillon-Goldstein’s (D.G.) Rho from the structural equation modelling (SEM).

Latent variable	R^2^_a_	Ave. Com.	D.G. Rho
Habitat quality		1.000	1.000
Soil conditions		0.558	0.747
Nutrient regulation	0.488	0.316	0.679
Biological control	0.378	1.000	1.000
Water quality	0.065	0.592	0.823
Freshwater supply	0.003	1.000	1.000
Food production	0.028	0.606	0.858
Raw materials	0.136	1.000	1.000
Aesthetic	0.043	1.000	1.000
Recreation	0.784	0.639	0.799
Environmental education	0.817	1.000	1.000
Carbon sequestration	0.259	1.000	1.000
**Relative Goodness of Fit (0.858)**			

**Fig 4:**
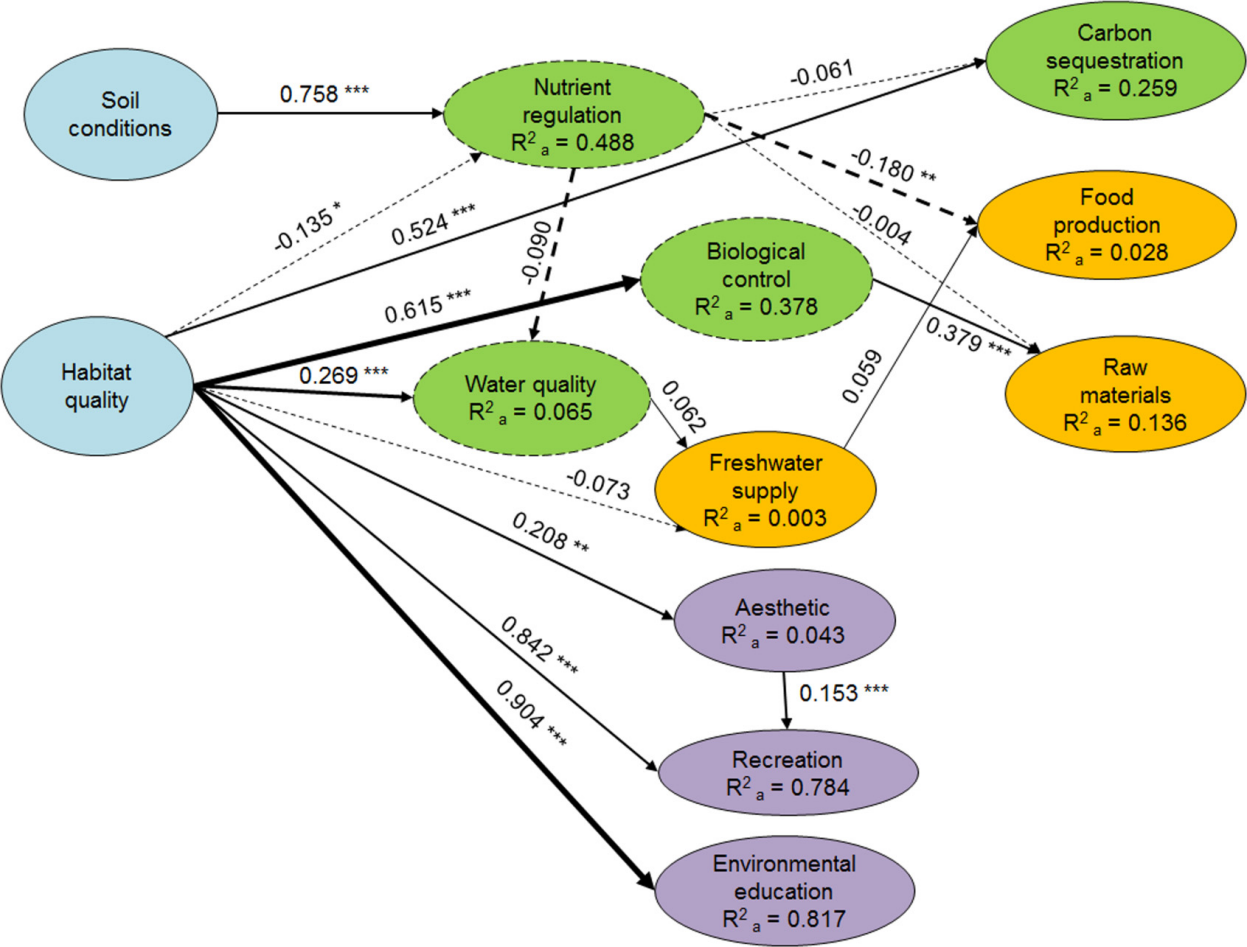
Structural equation model (SEM) results, showing the relationships between ecosystem services. Colours indicate the type of ecosystem service (green = regulating, gold = provisioning, purple = cultural) and supporting ecosystem properties (blue). Intermediate regulating services are dashed and final services are solid. Arrow thickness represents the percentage of the contribution to each service, where solid arrows represent positive relations and dashed arrows negative relations. Numbers near arrows indicate the standardized regression coefficient and the asterisks denote significance (* *p* ≤ 0.05; ** *p* ≤ 0.01; *** *p* ≤ 0.001).

The results of the SEM highlighted the fact that some ecosystem services were strongly related to others, while others were less influenced (Fig 4). Ecosystem properties (i.e., soil conditions and habitat quality) were key variables, given the significant influence they had on all ecosystem services to which they were related, except to freshwater supply. Soil conditions had a significant strong influence on nutrient regulation (β = 0.758). The largest influence of habitat quality was on environmental education (β = 0.904), recreation (β = 0.842), biological control (β = 0.615), and carbon sequestration (β = 0.524). Habitat quality also had significant but weak (β ≥ 0.1) influence on water quality and aesthetics, and a weak significant negative influence on nutrient regulation (β = -0.135). This latter could be explained by the different types of land uses included in the assessment, for which there might be opposite relationships. For instance, perennial forests have excellent quality (as assessed using the Riparian Quality Index [48]) but do not contribute much to nutrient regulation; conversely, cultivated land uses might have high concentrations of nutrients due to human inputs through fertilization. Intermediate services also had significant but weaker influence on final ecosystem services: biological control had a positive influence on raw materials (β = 0.379) and nutrient regulation had a negative influence on food production (β = -0.180), which can be explained by the fact that increasing food production can reduce nutrient regulation. Final services also displayed some interactions, namely, aesthetics had a weak significant positive influence on recreation (β = 0.153).

The contribution of each service is represented by arrow thickness in Fig 4 and highlights the main interactions maintaining the flow of ecosystem services. In our case study, supporting ecosystem properties (soil conditions and habitat quality) strongly influenced intermediate regulating services and cultural services, indicating that these are the key variables that mediate the flow of ecosystem services.

### Restrictions on the use of ecosystem services

**Fig 5:**
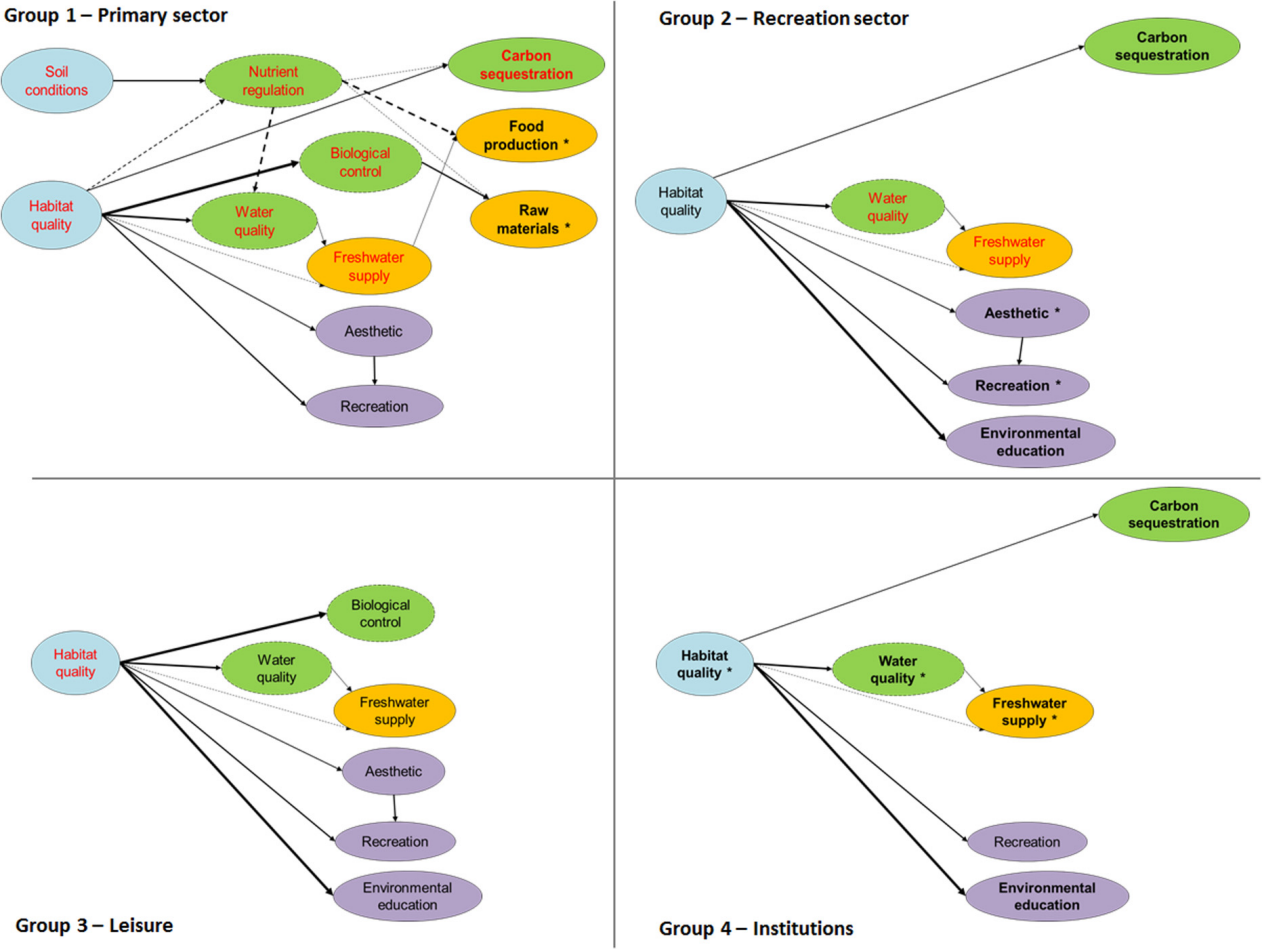
Ecosystem services related to each stakeholder group. Solid arrows represent positive relations between ecosystem services and dashed arrows negative relations. Colours indicate the type of ecosystem services (green = regulating, gold = provisioning, purple = cultural) and supporting ecosystem properties (blue). Intermediate regulating services are dashed and final services are solid. Impaired ecosystem services are in red, ecosystem services managed or co-produced are in bold, and they are marked with an asterisk (*) when managed by a single group. Note that habitat quality and carbon sequestration were only indirectly used by groups 1, 2 and 3, and that all ecosystem services linked to group 4 (excluding environmental education) were used indirectly.

Four stakeholders groups were identified (see Table 3): *primary sector*, *recreation sector*, *leisure* and formal *institutions*. The identification of the ecosystem services linked to each stakeholder group was useful to detect key ecosystem services for each stakeholder group in terms of their use, the ability of stakeholders to manage each service, and power asymmetries derived from the management of ecosystem services (Fig 5). The *primary sector* (Group 1) was linked to most ecosystem services, either by using, co-producing, or impairing them; in addition, they were the main managers of two provisioning services (raw materials and food production). The *recreation sector* (Group 2) used and impaired water-related services, and used and co-produced carbon sequestration and cultural services, part of which they had great ability to manage. *Leisure* (Group 3) was linked to cultural and water-related services and to biological control. These three groups were also indirectly linked to habitat quality. The ecosystem services linked to formal *institutions* (Group 4) were used indirectly, except environmental education, which was co-produced by a section of this group (i.e., the government bodies and scientists), and used by the other section (i.e., the schools and universities). Further, this group was the main manager of habitat quality, water quality, and freshwater supply.

These results together with a general overview of the power relationships among stakeholders enabled us to classify the ecosystem services of this case study within a rival/non-rival and excludable/non-excludable matrix (Table 5, see S3 File for details).

**Table 5:** Classification of the ecosystem services used in the River Piedra floodplain according to a rival/non-rival and excludable/non-excludable gradient.

	Excludable	↔	Non-Excludable
**Rival**	Food provision		
	Raw materials		
	Freshwater supply*		
	Aesthetic*		
	Recreation*		
	Environmental education*		
**Congestible ↕**	Soil conditions*		Water quality
	Nutrient regulation*		Soil conditions*
	Habitat quality*		Nutrient regulation*
			Habitat quality*
**Non-Rival**			Carbon sequestration
			Biological control
			Freshwater supply*
			Aesthetic*
			Recreation*
			Environmental education*

* Ecosystem services that can fall into several classifications according to specific situations. See main text for examples. Adapted from Fisher et al. [3]

### How stakeholders mediate access to ecosystem services

**Fig 6:**
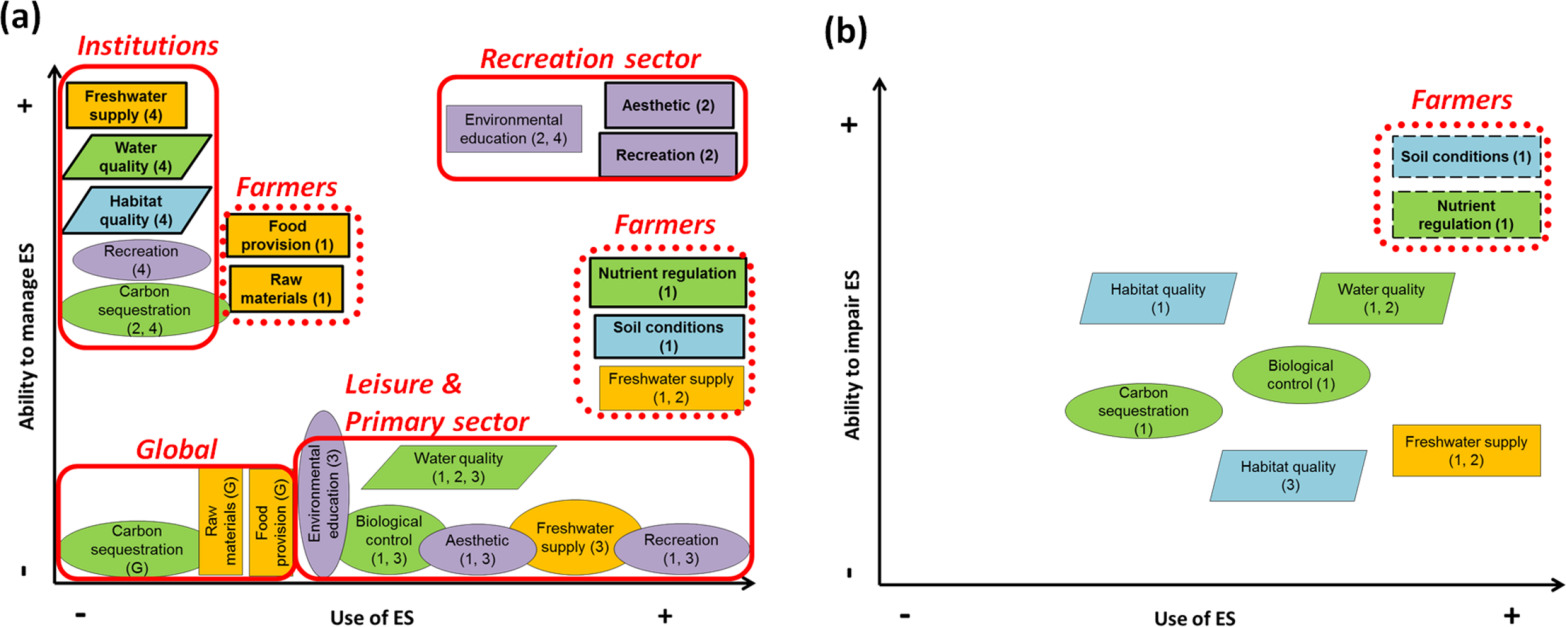
a) Stakeholders’ use *versus* ability to manage ecosystem services (ES), and b) Stakeholders’ use *versus* ability to impair ecosystem services (ES). The colour of the box indicates the type of ecosystem service (green = regulating, gold = provisioning, purple = cultural) and supporting ecosystem properties (blue). Rival and excludable services are in rectangles, non-rival and non-excludable services are in ellipses, and congestible services (non-excludable that can move from non-rival to rival) are in parallelograms. Bold boxes mark ecosystem services managed by a unique stakeholder group, and dashed boxes indicate ecosystem services used and impaired by the same single-stakeholder group. Numbers in parentheses indicate the stakeholder group (1 = Primary sector; 2 = Recreation sector; 3 = Leisure; 4 = Formal institutions; G = Global extent). The main clusters identified are marked in solid red boxes, and the secondary clusters in dotted red boxes.

We related stakeholders’ roles to the rival/excludable classification of ecosystem services by depicting each service in a diagram showing stakeholders’ use versus their ability to manage ecosystem services (Fig 6A) and stakeholders’ use versus their ability to impair ecosystem services (Fig 6B). The results on Fig 6A highlighted the effect of power relationships on access to ecosystem services and differentiated five types of clusters, which mostly corresponded to the previous stakeholder classification. Fig 6B provided complementary information especially useful to identify situations in which the same stakeholder group used and impaired the same ecosystem service.

We identified that formal *institutions* (Group 4) were the stakeholders with highest ability to manage and lowest use of ecosystem services (Fig 6A, top left corner). They managed a key supporting ecosystem property (habitat quality) and key intermediate regulating services (water quality) able to maintain the ecosystem services flow and, thereby, affect other stakeholders. Further, they managed final services, such as freshwater supply and recreation, without using them.

The *recreation sector* (Group 2) had the highest ability to manage and to use ecosystem services. This group contributed to produce cultural services by offering aesthetic enjoyment, recreation, and environmental education, exclusively managed some of these services, and also used them, benefiting from the tourism generated (Fig 6A, top right corner).

The *leisure* (Group 3) and *primary sector* (Group 1) were the stakeholders with the lowest or no ability to manage ecosystem services (Fig 6A, bottom right). Consequently, these stakeholders benefited from just the remaining non-excludable and non-rival ecosystem services (a part of cultural services, freshwater supply, water quality, and biological control).

Farmers from the *primary sector* (Group 1) were an intermediate cluster, as they had some opportunities to manage the services they used the most (nutrient regulation, soil conditions, and freshwater supply) through their farming practices (Fig 6A, right dotted line). However, this group impaired these same ecosystem services by overuse, driving non-rival ecosystem services to rival (Fig 6B, dotted line). Still in the second diagram, we observed that in the case of biological control, the use of the service did not directly imply degradation; rather this service was impaired by the farming practices used, which in turn may potentially affect other stakeholders. The impairers of habitat quality were *leisure* activities and the *primary sector*; however, they might not perceive it, as this service is not directly used but indirectly used through other services dependent on it. In addition, farmers were the unique producers of food and raw materials (Fig 6A, left dotted line). However, as these provisioning services are mostly exported outside the area, farmers just use them marginally (i.e., most of the production of such services is not on a self-consumption basis; rather, the income of these stakeholders mostly comes from the export of these goods).

Lastly, we identified a cluster that comprised those stakeholders having low use of ecosystem services and low ability to manage them. These were external to the ecosystem (e.g., the global food and raw materials markets and the carbon sequestration capacity of the atmosphere) (Fig 6A, bottom left).

### Key stakeholders control keystone ecosystem services: power matters

The identification of keystone ecosystem services to maintain the flow of services (Fig 4), together with the identification of the stakeholder groups that used and managed them (Fig 6) highlighted the critical effect of power asymmetries on access to ecosystem services. In our case study, the strongest power was held by the formal *institutions* group through the management of keystone supporting ecosystem properties and intermediate regulating services, on which many other services depend or that are used by most stakeholder groups. For instance, habitat quality had the strongest effects on the majority of ecosystem services: environmental education, recreation, and nutrient regulation (Fig 4). Additionally, water-related services (water quality and freshwater supply) were the most conflicting services as they aggregated the largest number of beneficiaries and impairers (Fig 6B). As a consequence, the *institutions* group had the power to promote synergies and trade-offs between ecosystem services and the power to limit the use of those services they managed to specific stakeholders (e.g., regulations on freshwater supply and fishing permits), excluding others, and thus, creating potential social imbalances.

Additionally, the *recreation sector* had strong power as they managed and used the cultural ecosystem services driving the economy of the area. Finally, the *primary sector* had intermediate but still important influence on some ecosystem services. For instance, they were able to moderately manage the provisioning services on which their main income is based (i.e., food production and raw materials). Interestingly, the SEM revealed that, in our case study, these services were fairly disconnected from other intermediate services (Table 4 and Fig 4) because they were mainly dependent on human inputs (e.g., irrigation and fertilizers) rather than on ecosystem functioning. Additionally, some farming practices impaired critical ecosystem services (e.g., habitat quality, soil conditions, and nutrient regulation) that determined the integrity of intermediate and final services, thereby creating powerful feedback to the stakeholders using those intermediate and final services.

## Discussion

### Potential and limitations of the analysis of social-ecological interactions along the ecosystem services flow

Integrating both ecological and social interactions along the flow of ecosystem services is key to understanding the likely asymmetries between stakeholders fostered by environmental management and to promoting sustainable management of ecosystem services [73]. Recent research has increasingly been addressing the flow of ecosystem services from production (supply-side) to use by society (demand-side) (e.g., [5,36,74,75]), and some have considered the implications of access to ecosystem services [76]. However, no framework has yet made explicit the existence of power relationships mediating both ecosystem services flows and stakeholder interactions. Specifically, recent research has pointed out the need to analyse the role of multiple stakeholder groups and their relationships with the provision, demand, and management of ecosystem services in order to contribute insights for sustainable management of ecosystem services [73]. In previous research, structural equation models have been used to test relationships between ecosystem properties and their effects on the provision of ecosystem services [77], and between ecosystem services and their effects on human well-being [78]. However, such analyses have not been previously connected to power relationships among stakeholders. Moreover, although Fisher et al. [79] discussed the importance of power related to poverty alleviation and ecosystem services, power relationships have still rarely been considered explicitly as a key factor determining asymmetries in the access to ecosystem services. Our study clearly demonstrates the relevance of power relationships in determining access to ecosystem services and its impact on the ecosystem services flow. Identifying and targeting such power relationships is essential for delineating environmental management policies while reducing trade-offs among ecosystem services [40] and thus, reducing social inequalities and conflicts.

The proposed framework was tested using our knowledge and data on the River Piedra case study and demonstrated its validity for uncovering three critical dimensions of ecosystem service actual supply: first, the dependence relationships among ecosystem services, and thus, the implications of their use and management; second, the formal power asymmetries between stakeholders; and third, the influence of such power relationships in the ability of stakeholders to access and to manage ecosystem services.

In addition, our work has been useful to test a methodology that identifies which stakeholders have the strongest power to mediate the flow of ecosystem services. These stakeholders were: (i) those controlling key ecosystem services (because they either can directly affect to other stakeholders, or can affect the capacity of the ecosystem to provide services); (ii) unique managers of a particular ecosystem service, and thus, able to control access and use of such service.

However, the application of such analyses in environmental management requires further work to specify and address informal and hidden power relationships between and within the stakeholder groups (e.g., social leadership, family ties, etc.). Other limitations for applying this study elsewhere are that it was time-consuming, as it required biological and social sampling; difficulties connecting biological sampling with social sampling to give insights into power relationships; and difficulties including the adjacent municipalities within the geographic boundaries (e.g., the river basin) but outside the targeted ecosystem (e.g. the river floodplain) in the power relationships analyses.

### The excludable and rival characteristics of ecosystem services

The classification of ecosystem services based on the concepts of excludability and rivalness (Table 5) concurred with most theoretical examples from Costanza [71] and Fisher et al. [3]. However, in our case study, we did not identify any examples of excludable but non-rival services, or non-excludable but rival, probably because management usually makes ecosystem services congestible (i.e., driving services from non-rival to rival). For instance, ecosystem services managed by the formal *institutions* group tended to fall into this category as this group can regulate the status of such services (e.g., policies to regulate water quality and waste water). Moreover, cultural services had two possible and opposite statuses: rival and excludable for activities performed on private sites or mediated by private companies (i.e., by the *recreation sector*), and non-rival and non-excludable, for those services enjoyed at open access sites. Hence, non-empowered stakeholders (i.e., the *leisure* group and the bulk of the *primary sector*) only had access to the remaining non-excludable and non-rival ecosystem services, and thus, are the most vulnerable stakeholders [72] at risk of being excluded from accessing the ecosystem services they need for their well-being.

These results contrast to other applications of this framework [80], indicating that the status of ecosystem services is highly context-dependent. In addition, classifying ecosystem services along the rival and excludable gradient enables accounting for the multiple possible statuses of an ecosystem service across land-use types and property rights. Indeed, the use of the land and access rights appeared as the critical factors determining the status of ecosystem services. Providing open access to lands and avoiding preventable dis-services (e.g., through conservation farming [81]) might change this classification. More importantly, focusing on managing the non-rival to rival movements of ecosystem services (i.e., the congestible services) would prevent their depletion.

### Against monopolization of ecosystem services: insights for environmental management

As the proposed framework pinpoints, environmental management mediates the use of ecosystem services, and thus, their interactions [82]. Additionally, our results enabled us to distinguish how the management of each ecosystem service and power relationships among stakeholders may condition its status across the rival/excludable matrix. For instance, single-stakeholder management systems in which ecosystem services are used and managed by a single stakeholder group generated positive feedback. Such positive feedback had two opposite effects: they either reinforced the service, for example, recreational activities managed by *the recreation sector* attracted more recreational activities; or depleted the service, such as for instance, soil conditions and nutrient regulation managed by the *primary sector* were consumed at faster rates than normal recovery; or the overuse of water by *leisure* activities impaired their initially good status. Although single-stakeholder management systems should ideally lead to negative feedback or internal self-regulation, there is a high risk of eliciting positive feedback, in which the service is depleted by unregulated use, decreasing the capacity of the system to supply services in the long term.

In addition, we identified top-down management strategies, where management is made from the higher levels of governance – usually involving stakeholders external to the social-ecological system – to the local population. This was the case for the formal *institutions* group that managed habitat quality and recreation, and the *recreation sector* that managed cultural services. These management systems did not foster potential synergies among the ecosystem services supplied by the River Piedra floodplain, such as enhancing habitat quality and cultural services [29], and neither strengthened the communities’ governance of their resources. Rather, the population in this area is mostly dependent on external capital such as the European Common Agricultural Policy subsidies for farmers or the investments made by the main companies in the recreation sector. Top-down management systems often have low resilience [83,84] and can fail to resolve resource-users’ conflicts [13] or rather contribute to create new environmental conflicts [85]. However, examples of participatory bottom-up management systems such as decentralized forest management in Tanzania [86], coastal ecosystems in Kenya [87], and estuaries in South Africa [88] have proved to be important to complement existing top-down systems. In our case study and similar rural areas, such participatory systems could be implemented by local governments and mediated by bridging institutions at different organizational levels, and should be adapted to the cultural and geographical characteristics of each social-ecological system. In fact, bridging organizational levels through nesting institutions is one of the basic principles that have been identified for creating robust bottom-up management systems, together with deliberation and participation of interested stakeholders, institutional diversity (i.e. formal and informal institutions) [89] and knowledge leadership [90]. Consequently, encouraging knowledge exchange through participatory mechanisms is important to guarantee that multiple stakeholders regularly interact and discuss about their interests, needs and management of particular ecosystem services [91].

## Conclusions

This paper shows that ecosystem services do not equally benefit the diversity of potential users, highlighting the importance of power relationships in ecosystem services’ interactions and their influence on the flow of ecosystem services. The dependency relationships between ecosystem services stressed the importance of the use and management of keystone ecosystem services, i.e., those services that are essential for the provision of either intermediate or final ecosystem services. We identified the formal power relationships exerted by stakeholders according to their ability to access and to manage ecosystem services, and the mechanisms they use to exert power. Therefore, those stakeholders able to manage such keystone ecological properties and ecosystem services can affect the well-being of other stakeholder groups by determining the ecosystem’s capacity to provide services and/or by controlling access to them.

Consequently, in order to delineate sustainable management practices that foster equal access to ecosystem services, it is necessary to contribute detailed information on: (i) ecosystem services’ interactions, (ii) the governance for each ecosystem service, (iii) the role of stakeholders regarding each ecosystem service, and (iv) the power relationships established among stakeholders. The present study presents a conceptual framework able to empirically operationalize the integration of such information.

## Supporting Information

S1 File Methods used to sample ecosystem services. (DOCX)

S2 File Stakeholders’ interviews. (DOCX)

S3 File Classification of ecosystem services along the rival/excludable gradient. (DOCX)
