# Supplementary material for: Ecosystem Services Flows: Why Stakeholders’ Power Relationships Matter
Source: PLoS One. 2015 Jul 22;10(7):e0132232. doi: 10.1371/journal.pone.0132232 (PMC4511803; doi:10.1371/journal.pone.0132232)
Supplement: S1 File — (DOCX) [file pone.0132232.s001.docx]

**S1. Methods used to sample ecosystem services.**

**Habitat quality**

**Riparian Quality Index:** We used the Riparian Quality Index (RQI) (González del Tánago and García de Jalón, 2011) to assess habitat quality. The RQI evaluates seven riverbank attributes (i) dimensions of land with riparian vegetation (average width of riparian corridor); ii) longitudinal continuity, coverage, and distribution pattern of riparian corridor (woody vegetation); iii) composition and structure of riparian vegetation; iv) age diversity and natural regeneration of woody species; v) bank conditions; vi) floods and lateral connectivity; and vii) substratum and vertical connectivity), providing a relative score between 10 and 120 that was reclassified from 0 to 100. RQI was estimated in three plot replicates by land use type (i.e., dry cereal crops, irrigated cereal crops, poplar groves, fruit groves, riparian forests, abandoned crops, and urban areas) between July 2011 and July 2012.

**Soil conditions**

**Soil formation:** We measured the organic matter content in topsoil (0-10 cm) as an indicator of this service in July 2011 and July 2012. We sampled three patch replicates in each of the seven main land uses of our study area except in urban areas, where most of the soils are sealed (Felipe-Lucia et al., 2014). Three transects perpendicular to the river channel were established in each patch and three samples along each transect were taken at 1 m, 5 m, and 15 m away from the river. Half a kilogram of topsoil was collected at each point, dried (48 hours at 60ºC), sieved and milled. Total organic matter was analyzed using the LOI protocol (Lost On Ignition, Nelson and Sommers, 1996) and the average value (as soil weight percentage) of the two years was used as a measure of each sampling point.

**Soil stability:** We recorded the organic matter layer thickness in topsoil (0-10 cm) as an indicator of this service (Daily, 1997). We sampled three patch replicates by land use type except in urban areas, where most of the soils are sealed (Felipe-Lucia et al., 2014). Three transects perpendicular to the river channel were established in each patch and three measurements along each transect were taken at 1 m, 5 m, and 15 m away from the river. The organic matter layer depth (cm), excluding leaf litter, was recorded in the field with a measuring tape in July 2011 and July 2012, and average data of each point across both years were used as an indicator.

**Water quality**

**Nitrite content in water (NO_2_^-^):** We analyzed the nitrite content in water (ppm) as a measure of pollutant concentration in the river. 21 samples were collected monthly along the river in 2009. The sampling was designed to cover a wide range of situations representing the water quality of the study area and was repeated in specific months of 2010 and 2011 to account possible variation in the water flow rates. Samples were kept refrigerated and analyzed in laboratory within a week using standard methods (i.e., ionic chromatography (APHA, 1998)). Values per sample point were averaged across years and then by municipality. Because nitrite content in water is an indicator of pollutant concentration, we used the inverse values to account for water quality.

**Nitrate content in water (NO_3_**^-^**):** We analyzed the nitrate content in water (ppm) as a measure of pollutant concentration in the river. 21 samples were collected monthly along the river in 2009. The sampling was designed to cover a wide range of situations representing the water quality of the study area and was repeated in specific months of 2010 and 2011 to account possible variation in the water flow rates. Samples were kept refrigerated and analyzed in laboratory within a week using standard methods (i.e., ionic chromatography (APHA, 1998)). Values per sample point were averaged across years and then by municipality. Because nitrate content in water is an indicator of pollutant concentration, we used the inverse values to account for water quality.

**Phosphate content in water (PO_4_**^-^**):** We analyzed phosphate content in water (ppm) as a measure of pollutant concentration in the river. 21 samples were collected monthly along the river in 2009. The sampling was designed to cover a wide range of situations representing the water quality of the study area and was repeated in specific months of 2010 and 2011 to account possible variation in the water flow rates. Samples were kept refrigerated and analyzed in laboratory within a week using standard methods (i.e., ionic chromatography (APHA, 1998)). Values per sample point were averaged across years and then by municipality. Because phosphate content in water is an indicator of pollutant concentration, we used the inverse values to account for water quality.

**Nutrient regulation**

**Total carbon in top soil (C):** We measured the total carbon content in topsoil (0-10 cm) as an indicator of this service in July 2011 and July 2012. We sampled three patch replicates in each of the seven main land uses of our study area except in urban areas, where most of the soils are sealed (Felipe-Lucia et al., 2014). Three transects perpendicular to the river channel were established in each patch and three samples along each transect were taken at 1 m, 5 m, and 15 m away from the river. Half a kilogram of topsoil was collected at each point, dried (48 hours at 60ºC), sieved and milled. Total carbon was measured using a macro elemental analyzer (Vario Macro Max CN) and results were expressed in concentration (ppm). The average value (as soil weight percentage) of the two years was used for each sampling point.

**Total nitrogen in top soil (N):** We measured the total nitrogen content in topsoil (0-10 cm) as an indicator of this service in July 2011 and July 2012. We sampled three patch replicates in each of the seven main land uses of our study area except in urban areas, where most of the soils were sealed (Felipe-Lucia et al., 2014). Three transects perpendicular to the river channel were established in each patch and three samples along each transect were taken at 1 m, 5 m, and 15 m away from the river. Half a kilogram of topsoil was collected at each point, dried (48 hours at 60ºC), sieved and milled. Total Nitrogen was measured using a macro elemental analyzer (Vario Macro Max CN) and results were expressed in concentration (ppm). The average value (as soil weight percentage) of the two years was used for each sampling point.

**Total phosphorus in top soil (P):** We measured the soluble reactive phosphorus (SRP) content in topsoil (0-10 cm) as an indicator of this service in July 2011 and July 2012. We sampled three patch replicates in each of the seven main land uses of our study area except in urban areas, where most of the soils are sealed (Felipe-Lucia et al., 2014). Three transects perpendicular to the river channel were established in each patch and three samples along each transect were taken at 1 m, 5 m, and 15 m away from the river. Half a kilogram of topsoil was collected at each point, dried (48 hours at 60ºC), sieved and milled. SRP was extracted following the Olsen protocol (Olsen et al., 1954) and filtered. The extract was analyzed in an ionic chromatograph. The average value (as soil weight percentage) of the two years was used for each sampling point.

**Biological control**

**Vertical vegetation structure:** To estimate the richness of vertical vegetation structure we surveyed three plot replicates by land use type in July 2012. Urban areas were excluded as soils are sealed. Within each plot, three floodplain-wide transects (average transect length 57 m) perpendicular to the river channel were established 25 m apart. In each transect, we used the point-intercept method (Goodall, 1952) every 10 cm to estimate species occurrence and percent cover of each plant species (i.e., number of contacts relative to the total number of points sampled). Identification of plants at the genus or species level was corroborated using a regional herbarium (namely, herbarium of Jaca: http://proyectos.ipe.csic.es/herbario‎) and a botanist expert. Then, we classified vegetation records into four types of vertical vegetation structure (namely, herb, creeper, shrub, and tree) and estimated the richness of plants strata using the vegan package (Oksanen et al., 2013) of the R software (R Development Core Team, 2013).

**Carbon sequestration**

**Carbon sequestration:** We used carbon (CO_2_) sequestration of woody plants as a surrogate of this service (Trabucchi et al., 2014). Annual CO_2_ sequestration rates by land use type were obtained from a national database (Montero et al., 2005; CITA, 2008) which estimated the amounts of carbon stored by above- and below-ground biomass of the main Spanish plant species and woody formations. Calculations are based on the species annual growth and transformed into CO_2_ equivalent tons per hectare using stoichiometric equations (Montero et al., 2005). We used data from the closest plant species or woody formations to the land cover composition of our study area (e.g., average data of apple, pear, peach, and plum groves for fruit groves). Herbaceous species –and therefore, irrigated cereal crops and dry cereal crops – were not included because their annual CO_2_ storage balance is null (CITA, 2008); for abandoned crops, only its woody formations (e.g., hawthorn) were considered. Urban areas have not been included either, since they act usually as a source of carbon rather than as a sink (but see Davies et al., 2011).

**Freshwater supply**

**Water consumption:** Water concessions of the River Piedra were obtained from public data provided by the water management body (Confederación Hidrográfica del Ebro, <http://iber.chebro.es/webche/raInfo.aspx>, accessed on 2011). Calculations were made to obtain the annual cubic meters supply at each municipality, which was used as an indicator.

**Food production**

**Yield:** We estimated the average yield (kilograms per hectare) of each of the main land use types of our study area from the latest update of a national public database (Instituto Nacional de Estadística, updated on 30.10.2012). We averaged irrigated wheat, barley, and corn yields to estimate food provision by irrigated crops; dry wheat, barley, and corn yields for dry cereal crops; and apple, pear, peach, and plum grove yields for fruit groves. The other land uses were assigned a yield value of 0.

**Calories:** Yield values for the crops growing within the study area were obtained from national databases statistics (Instituto Nacional de Estadística, updated on 30.10.2012), expressed as kilograms per hectare, and multiplied by the crop caloric value (kilocalories per 100 grams). The ecosystem service value is expressed as kilocalories per hectare (Felipe-Lucia et al., 2014).

**Gross value:** Crops productivity was calculated based on crops yield (Felipe-Lucia and Comín, 2015) and the index of agricultural prices provided by the regional government (Gobierno de Aragón, <http://www.aragon.es>).

**Raw materials:**

**Production:** We used the yearly aboveground dry biomass accumulation by land use type as a measure of the raw materials production. Values were obtained from a national database (Montero et al., 2005; CITA, 2008) that estimated the annual growth rates of woody species as tons of dry biomass per hectare, according to the average timber diameter. We adapted data from the closest woody species to the land cover composition of our study area (e.g., average data of apple, pear, peach, and plum groves for fruit groves). Herbaceous species –and therefore, irrigated cereal crops and dry cereal crops– were not included because their annual accumulated biomass balance is null (CITA, 2008), whereas for abandoned crops, only its woody formations (e.g., hawthorn) were considered. Note that biomass production is an indicator of the potential biomass provision by each land use type, thus referring to the potential use of the biomass as a raw material (i.e., making this use of the land incompatible with the provision of other services such as fruit production or carbon sequestration).

**Aesthetics**

**Pictures:** We counted the number of different people uploading pictures to Panoramio^[[1]](#footnote-1)^ from each of the main land uses of each municipality within the floodplain of River Piedra. We used the finest resolution to count each single picture. This measure has been considered to be more appropriated than the total number of pictures, which would rather reflect the individual activity of photographers (Casalegno et al. 2013). Pictures focusing on buildings from all sorts (e.g., houses, towers, crosses, churches, hermitages, monasteries, etc.) were not considered because they were not directly related to any use of the ecosystem. The platform was accessed on 27.03.2014.

**Recreation**

**Fishing:** Available fishing stretches for recreational use at the River Piedra were obtained from the fishing regulatory policy of 2012 for the Autonomous Community of Aragon (BOA 2012) and drawn using GIS tools (ArcGIS 10.0, ESRI). Fishing available stretches were computed for both riversides. Stretches were converted into polylines, their perimeters calculated and summarized into stretches available or unavailable for fishing. Polylines were converted into polygons and intersected to the land use cover with a buffer of 10 m to add both the land use and municipality information of each stretch of the river. Then lengths were recalculated. The length of the river in each land use type of each municipality in relation to the total length of the river (i.e., including areas unavailable for recreational fishing) and in reference to a 1 hectare patch (a patch of 100 meters of side) was used as an indicator (i.e., Fishing at land use A = (Total length of land use A / Total length of the river) x 100) (Felipe-Lucia et al., 2014)).

**Sports:** Tracks of post-signed and user-designed paths were downloaded from both the regional tourist office website and wikilocs (<http://senderos.turismodearagon.com> and [www.wikiloc.com](http://www.wikiloc.com), respectively; accessed on: 12.10.2012) following Trabucchi et al. (2014). Tracks within the study area were unified using GIS tools (QGIS, Quantum GIS Development Team), and overlapped to the study area viewshed. Then the viewshed of the shapefile obtained was calculated and intersected to the land use cover. Finally the extent of each land use that can be seen from the open-to-public used paths was calculated. Average values per hectare of each land use at each municipality were used as an indicator (Felipe-Lucia et al., 2014).

**Picnic areas:** We used the number of areas used for social amenity (e.g., picnic areas) within the study area as an indicator of this service (Posthumus et al., 2010). The number of areas in each municipality was counted by land use type in August 2012. To keep spatial scale consistency, these data were transformed into a density measure (i.e., Total number of picnic areas by land use type and municipality / Land use type cover extent at each municipality) (Felipe-Lucia and Comín, 2015).

**Environmental education**

**Information panels**: We used the number of panels with information about the ecosystem as an indicator of this service. This was the only available indicator distinguishing among land use types. Panels were counted in each municipality by land use type in August 2012. To keep spatial consistency, these data were transformed into a density measure (i.e., Total number of panels by land use type and municipality / Land use type cover extent at each municipality) (Felipe-Lucia and Comín, 2015).

**Additional references for S1**

Centro de Investigación y Tecnología Agroalimentaria de Aragón/Agrifood Research and Technology Centre of Aragon (CITA), 2008. Estudio sobre la funcionalidad de la vegetación leñosa de Aragón como sumidero de CO2: existencias y potencialidad (estimación cuantitativa y predicciones de fijación). Centro de Investigación y Tecnología Agroalimentaria de Aragón, Zaragoza, Spain. Retrieved from: <http://www.aragon.es/estaticos/GobiernoAragon/Departamentos/MedioAmbiente/Areas/03_Cambio_climatico/06_Proyectos_actuaciones_Emisiones_GEI/estudio.pdf>

Daily, G.C., 1997. Nature’s services: societal dependence on natural ecosystems. Island Press, Washington, DC.

Davies, Z.G., Edmondson, J.L., Heinemeyer, A., Leake, J.R., Gaston, K.J., 2011. Mapping an urban ecosystem service: quantifying above-ground carbon storage at a city-wide scale. Journal of Applied Ecology 48, 1125–1134. doi:10.1111/j.1365-2664.2011.02021.x

Environmental Systems Resource Institute (ESRI), 2012. ArcGIS Desktop (10.0). Redlands, California, USA.

Felipe-Lucia, M.R., Comín, F.A., Ecosystem services-biodiversity relationships depend on land use type in floodplain agroecosystems. Land Use Policy; Vol. 46C, pp. 201-210. doi: 10.1016/j.landusepol.2015.02.003

Oksanen, J., Blanchet, F.G., Kindt, R., Legendre, P., Minchin, P.R., O'Hara, R.B., Simpson, G.L., Solymos, P., Stevens, M.H.H., Wagner, H., 2013. vegan: Community ecology package. R package version 2.0-7. <http://CRAN.R-project.org/package=vegan>

Olsen, S.R., C.V. Cole, F.S. Watanabe, Dean, L.A., 1954. Estimation of available phosphorus in soils by extraction with sodium bicarbonate. USDA Circular 939:1-19. Gov. Printing Office Washington D.C.

Posthumus, H., Rouquette, J.R., Morris, J., Gowing, D.J.G., Hess, T.M., 2010. A framework for the assessment of ecosystem goods and services; a case study on lowland floodplains in England. Ecol. Econ. 69, 1510–1523. doi:10.1016/j.ecolecon.2010.02.011

Quantum GIS Development Team, 2012. Quantum GIS Desktop (1.8.0). Open Source Geospatial Foundation Project, Beaverton, Oregon, USA. <http://qgis.osgeo.org>

R Development Core Team, 2012. R: a language and environment for statistical computing. R Foundation for Statistical Computing, Vienna, Austria. <http://www.R-project.org/>

1. Panoramio is a web platform supporting pictures having a special focus on landscape and environment. This platform has already been utilized in previous research about social preferences on ecosystem services (Casalegno et al., 2013). [↑](#footnote-ref-1)
