# Supplementary material for: Ecosystem Services Flows: Why Stakeholders’ Power Relationships Matter
Source: PLoS One. 2015 Jul 22;10(7):e0132232. doi: 10.1371/journal.pone.0132232 (PMC4511803; doi:10.1371/journal.pone.0132232)
Supplement: S2 File — (DOCX) [file pone.0132232.s002.docx]

**S2. Stakeholders’ interviews.**

**Table A. Description of the respondents quoted in Table S2.2. Note that only a selection of the total sample is showed.**

| **Code (Group_ intervieew)** | **Group** | **Function** | **Interview date** | **Interview length (h:mm)** |
| --- | --- | --- | --- | --- |
| 1_1 | Primary sector | Local farmer, retired | 30.08.2011 | 1:07 |
| 1_2 | Primary sector | Local shepherd | 30.08.2011 | 1:31 |
| 1_3 | Primary sector | Local farmer | 06.08.2011 | 1:22 |
| 1_4 | Primary sector | Local farmer | 23.03.2012 | 1:01 |
| 1_5 | Primary sector | Local farmer | 31.08.2011 | 1:11 |
| 1_6 | Primary sector | Local farmer | 31.08.2011 | 1:34 |
| 1_7 | Primary sector | Local shepherd, retired | 03.08.2011 | 0:30 |
| 2_1 | Recreation sector | Local lodge owner | 30.08.2011 | 0:35 |
| 2_2 | Recreation sector | Local nature business | 01.08.2011 | 0:58 |
| 2_3 | Recreation sector | Camping owner | 26.08.2011 | 1:05 |
| 2_4 | Recreation sector | Local hotel owner | 04.08.2011 | 0:45 |
| 2_5 | Recreation sector | Adventure enterprise partner | 2.10.2011 | 0:39 |
| 2_6 | Recreation sector | Local lodge owner | 30.08.2011 | 1:47 |
| 3_1 | Leisure | Local seasonal resident (weekends, holidays, etc.) | 03.08.2011 | 2:40 |
| 3_2 | Leisure | Local seasonal resident (weekends, holidays, etc.) | 07.03.2012 | 1:14 |
| 3_3 | Leisure | Local seasonal resident (weekends, holidays, etc.) | 01.08.2011 | 1:04 |
| 3_4 | Leisure | Seasonal resident (weekends, holidays, etc.), retired | 02.08.2011 | 0:54 |
| 3_5 | Leisure | Local seasonal resident (weekends, holidays, etc.) | 28.09.2011 | 0:31 |
| 3_6 | Leisure | Permanent resident, retired | 5.10.2011 | 1:15 |
| 4_1 | Institutions | Environmental technician (engineer) working on several environmental projects on the area (e.g., bioengineering) | 20.02.2012 | 0:50 |
| 4_2 | Institutions | Local council, councillor | 05.08.2011 | 1:00 |
| 4_3 | Institutions | Local council, mayor | 05.08.2011 | 1:00 |
| 4_4 | Institutions | Elementary school teacher | 05.10.2011 | 1:14 |
| 4_5 | Institutions | High school teacher | 05.10.2011 | 0:59 |
| 4_6 | Institutions | University professor | 22.02.2012 | 1:23 |
| 4_7 | Institutions | Local council, mayor | 05.08.2011 | 0:30 |

**Table B. Ecosystem services co-produced, used, and impaired by each stakeholders group.** Citations are in italics, and numbers in parentheses indicate the code of the interviewee, described in Table S2.1. Acronyms: CHE=Confederación Hidrográfica del Ebro (Regional water management body of the Ebro basin); MMA=Ministerio de Medio Ambiente (Ministry of the Environment).

| **Ecosystem service** | **Stakeholder group** | **Co-produced by** | **Used by** | **Impaired by** |
| --- | --- | --- | --- | --- |
| *Soil conditions* | 1- Primary sector |  | Famers use soil properties to growth their crops. For instance, they benefit from organic matter content and layer’s thickness.  *"Soil is the most important, when it is flooded gives 25% more yield the next year. It is good for farming because the sediments are good*“ (3_1) | Farmers’ practices (e.g., tillage) erode soils by oxidizing the organic matter and breaking soils’ structure.  "*Now they [the farmers] use herbicides. They say everything is cleaner, but it breaks riverbanks and the land crumbles*” (1_2) |
| *Habitat quality* | 1- Primary sector |  | [Used indirectly] | Usually farmers prefer clear riverbanks to avoid shading diminishing crops yield. In consequence, they used to cut trees and avoid new planting. “W*e used to clean the riverbanks, we cut tree branches and the grass*” (1_3) *"We do not want big trees because they do not allow crops to grow up, and we need yields to make money. It’s our job, and some years are good and some others are worse"* (1_5) “*If we clean often we also destroy the river ecological system, because there used to be pools but now they are clogged*” (4_2) |
|  | 2- Recreation sector |  | [Used indirectly] |  |
|  | 3-Leisure |  | [Used indirectly] | Some fishermen leave waste behind close to fishing areas, and break trees’ branches to access the river.  "*People are not aware of the value we have here. There is a lack of awareness and environmental education*” (3_2) |
|  | 4-Institutions | Scientists, technicians and the government contribute to enhancing this service by ecological restoration projects. "… *yes, protection of the aquifer, and the landscape is possible and it has to come from the CHE and the MMA*” (3_2) "*The project was ordered by the MMA and the CHE, so the most important issues here were water, soils, plants, and animals. Productive uses are not so important, neither tourism. Also the landscape, educational issues, social relationships, and life quality. That would be the scheme for the MMA, which I followed"* (4_1) | [Used indirectly] |  |
| *Water quality* | 1- Primary sector |  | The fish farm benefits from the quality of the water sources of the River Piedra, which makes trout to grow up better than in other rivers. "*The water of our river is so good that the fish farm grows here the juvenile fishes. Each week, a lorry collect 25000 kg of trout and go to another place to grow up to fatten*" (3_3) | Farmers impair water quality because of diffuse pollution and crops’ run-off. The fish farm pollutes the water by direct discharge.  *"Water has such an anise color. It was clear before, but now it is waste water from the fish farm and Cimballas' houses, and some other chemical fertilizers (…) Last year I drank water from the river and I had to stay in bed for three days, with gastroenteritis, now you cannot drink water from any place"*(3_1) *"There were not crops, nitrates, sulphates, etc. We have destroyed the river, because the easiest thing it is to use chemical herbicides....but if the Ministry of Agriculture allows us to use it...and also the fish farm of Cimballa, it had not wastewater treatment plant and discharged some wastewater into the river"* (1_6) |
|  | 2- Recreation sector |  | They benefit from performing activities in a clean water river, which attract tourists to come.  "*The economy of this area depends upon the river, the Monasterio de Piedra, the spa resorts, and also most of the people depend on them*" (4_4)  "*It’s trendy to attract rafting and kayaking tourists*” (1_2)  "*The main attraction is the Monasterio de Piedra, but there are also the sources of the River Piedra (…) and the reservoir (…)*" (2_1) | Hotels impair water quality by the discharge of untreated wastewater. Their daily activities pollute water because of the lack of functional wastewater treatment plants in the villages, which spill out directly to the river. This fact worsens during summer months, when population doubles and the water flow decreases, giving as a result a bad colored and smelly river.  *"In LLumes there is any waste water collection, so everything goes directly into the river and it can be dangerous because we are also drinking from wells*" (1_4)  *“Because the waste water from Carenas spills directly to the river and in Castejón, too… and then if the river water flow is low it smells badly”* (4_7) |
|  | 3-Leisure |  | Tourists benefit from having a clean water river to enjoy leisure activities (e.g., relaxing, kayaking, and fishing).  "*This stretch of the river is really good, I do not see dirty water, I see very clear water"* (4_2) |  |
|  | 4-Institutions | The central government is in charge of providing wastewater treatment plants to villages, and local governments are in charge of assuring their proper functioning. The CHE should care about its quality. *"The wastewater collection has been demanded several years ago, but nobody does a thing. We are systematically mistreating the river*" (1_4) | [Used indirectly] |  |
| *Nutrient regulation* | 1- Primary sector |  | Famers benefit from natural nutrient regulation to growth their crops. For instance, they benefit from organic carbon, nitrogen, and phosphorus content.  "*Soil is the most important, when it is flooded gives 25% more yield the next year. It is good for farming because the sediments are good*” (3_1) | Farmers’ practices (e.g., tillage) erode soils by oxidizing the organic nutrients. Additionally, the use of chemical nutrients deregulates natural nutrient cycling.  *“Now they [the farmers] use chemical fertilizers and before they used manure”* (3_3) |
| *Biological control* | 1- Primary sector |  | Farmers benefit from natural biological controllers such as birds and other insects.  *“There were understory formations that hosted many different bird species and also snakes and other animals”* (1_2) | Farmers impair this service because of the use of chemical herbicides and other pesticides which are not specifically targeted for undesirable species for farming, thus, affecting the natural regulation of the ecosystem. *"Now they [the farmers] use herbicides. They say everything is cleaner, but it breaks riverbanks and the land crumbles*" (1_2).  "*There were not crops, nitrates, sulphates, etc. We have destroyed the river, because the easiest thing it is to use chemical herbicides....but if the Ministry of Agriculture allows us to use it...*." (1_6) |
|  | 3-Leisure |  | People benefit from natural biological controllers such as birds and insects eating mosquitos and crops’ pests.  “*Here there are not many mosquitos because the temperature of the water controls that, and here the water is fresh*”(3_4) |  |
| *Carbon sequestration* | 1- Primary sector | Fruit groves and poplar groves owners could contribute to this service by the amounts of carbon sequestered by their trees.  *“We are planting our lot with walnut trees (…) others have poplars”* (3_2) |  | Farmers’ practices (e.g., tillage, use of chemical pesticides, etc.) can liberate carbon to the atmosphere. However, the potential harm caused by farmers is just a little contribution to the global problem, which means farmers do not directly affect other users but rather they contribute to the general degradation of this service.  [Not perceived by the interviewees] |
|  | 2- Recreation sector | Tree plantations made by the Monasterio de Piedra a hundred years ago contribute to carbon sequestration.  [Unrecorded communication] |  |  |
|  | 4-Institutions | Ecological restoration projects comprising tree plantations (financed by the MMA and assessed by scientists and technicians) contribute to carbon sequestration.  *“They [the MMA] should do more plantations to have more trees”* (1_7) | [Used indirectly] |  |
| *Food provision* | 1- Primary sector | They produce food from crops and trout farming.  “*There are self-consumption, and some professional farmers*” (2_2) |  |  |
| *Raw materials* | 1- Primary sector | Poplar groves owners produce wood.  *“Poplars used to be cut down and the wood was used to build houses*” (3_1) |  |  |
| *Freshwater supply* | 1- Primary sector |  | Farmers benefit from access to water from the river and ditches. The fish farm takes freshwater directly from a source.  “*The waterwheels allow to divert water to the ditches”* (2_1)  “*I have irrigated lands and turn dry lands into irrigated with the water from the river”* (1_4)  “*All the water used by the fish farm comes from the sources*” (2_3) | The fish farm has exclusive use of a water source, impeding other users to access to it. Water demand by farmers out of the catchment decreases the amount of water remaining in the river for other uses.  “*There is another source close to the road (…) there was a waterfall (…) but the fish farm built a wall and now it is stored (…). You can still see the water, but it is not so beautiful*” (1_5)  “*When the reservoir was built, everybody had to emigrate (…), and now the farmers from La Almunia, are benefiting from that richness, (…) and control the reservoir and we can do nothing*” (3_3) |
|  | 2-Recreation sector |  | Hotels use the water for domestic use. Adventure companies benefit from high flows to perform their activities in the river. The Monasterio de Piedra also benefits from higher flows increasing the aesthetic impact of the waterfalls. *“With the idea of having higher water flow and less water loss... the river is channelized and then there is more water in the Monasterio de Piedra waterfalls (…) 90% of the River Piedra is currently used to supply water to the waterfalls of the Monasterio de Piedra. They are nothing without water and with water they are a company making a lot of money. They are a private company using a public good -the water- to make money*” (1_4) |  |
|  | 3-Leisure |  | The high flow levels benefit clients of adventure companies.  *“The River Piedra has a large and continuous flow; even when there are droughts here there is always water”* (1_3) |  |
|  | 4-Institutions | The CHE regulates the use of the water.  *"… and then they [the CHE] cut down the river, by the end of September they close the dam and the river flows with a very little water*” (1_7)  *“They [the CHE] cut down the release from the reservoir and the water flow in the river is left at a minimum level, which is too low”* (4_7) | [Used indirectly] | The CHE entitles to the use of water, which can create disagreements or inequalities among users.  *“In Castejón and Carenas we have a concession (from the CHE) of free water for irrigation”* (1_7)  *“We could irrigate more lands but the CHE does not give more concessions”* (1_4)  *"The irrigation function of the river, how is it? it is how downstream people wants: the water retained in the reservoir"* (1_2) |
| *Aesthetics* | 1- Primary sector |  | They enjoy the beauty of the place where they live. "*The water source of the "eyes" is wonderful. And it is so deep, more than 5 meters. It is so wonderful to see how the water comes up gushing*" (1_5). |  |
|  | 2- Recreation sector | The Monasterio de Piedra contributes to this service by the maintenance and enhancement of the waterfalls and tree cover.  *“They [the Monasterio de Piedra] diverted the water flow to have more water in the waterfalls”* (1_4) | The aesthetics value of the area is the main attraction for tourists. "*People likes it, it is a natural park very nice, people loves it... you come here and you see a natural park with waterfalls..."* (2_4) |  |
|  | 3-Leisure |  | They enjoy the beauty of the place where they perform their leisure activities "*The River Piedra has many charming beauty spots*" (3_1)  *"Look at the picture with the watermill, you'll see what a wonderful landscape makes the river... look this riverine landscape.... we have many pictures of the river and the bridge in the brochures of the village festival”* (4_3) |  |
| *Recreation* | 1- Primary sector |  | Local people use the area for personal recreation. "*The fact that you can go for a walk along the river every morning or every afternoon is pure enjoyment, and it is only appreciated by those loving nature.*" (3_3) |  |
|  | 2- Recreation sector | They offer recreational activities *“The project is about small rafting boats and kayaks, maybe hydrospeed, (…) and also climbing in Nuévalos”* (2_5) | They benefit from having clients visiting the area for recreational activities. "*People likes it, it is a natural park very nice, people loves it... you come here and you see a natural park with waterfalls..."* (2_4)  *“I walk many, many, many days in the afternoon by the riverside with my dogs. It’s a very pleasant walk, fresh without sun beats, and very good”* (2_4) |  |
|  | 3-Leisure |  | The main reason of visiting the area is for performing recreational activities (e.g., sports, fishing, picnicking). *"People like going for a walk along the river, people likes it, and then we have a reservoir, a picnic area, we have a very beautiful landscapes here, I like them. Yes, it is important because it is a beautiful landscape and people comes and watch it but it gives any money to the village; it is more a moral benefit."* (4_3)  *“There is a place for bird watching, if you like, you can enjoy the landscape, the architecture, oenology, customs, etc.”* (3_6) |  |
|  | 4-Institutions |  | [Used indirectly] | Some fishermen agree that several actions performed by the CHE have caused a decrease in trout. *"The fisheries were destroyed when they [the CHE] cleaned the river, they destroyed all the fisheries and now there are some trout, but only a few"* (1_1)  *"There is no more sand where it used to be; now there is only that black mud… and trout is very delicate and if it is not good she doesn't raise juveniles...*" (2_4) |
| *Environmental education* | 2- Recreation sector | Nature/adventure companies usually are pro-environmental education, so producing the service. The Monasterio de Piedra has many panels providing information about the functioning of the ecosystem.  *“They [the companies] explain the uses of the water in the River Piedra, the trees, the birds, and many other things”* (3_6) | Nature/adventure companies usually are pro-environmental education and use the educative panels and other facilities.  *“Now there is a sighting hut and some panels with the birds we can observe from here”* (2_2) |  |
|  | 3-Leisure |  | People can learn from the educative panels.  *“Now there is a sighting hut and some panels with the birds we can observe from here”* (2_2) |  |
|  | 4-Institutions | Local councils and the MMA (advised by scientists) have provided the area with panels that explain the ecosystem functioning of the area  “*We have created a rest area close to the road and from there you can see a wonderful landscape of the valley"* (4_3) | Scholars and scientific groups visit the area to learn about the ecosystem and benefit from the existing panels. "*We teach about trees, learning their names, making the difference among them and their fruits.... we also do orienteering, trekking, and even clean the river and learn to respect the river*" (4_4) "*In the Piedra valley what is important is geology. There are huge ripples formations, fossils around Carenas and Castejón. And then in the gorges you have old hives, etc."* (4_5) *"Torralba has been more promoted and people from the university has come to visit the gorges and surroundings"* (3_5)  “*We have many publications about the River Piedra. We have investigated fossil and current tufa since the last 12 years. And we teach about it at the University. We have also organized a conference where we visited the area”* (4_6) |  |
|  |  |  |  |  |
