# Supplementary material for: Ecosystem Services Flows: Why Stakeholders’ Power Relationships Matter
Source: PLoS One. 2015 Jul 22;10(7):e0132232. doi: 10.1371/journal.pone.0132232 (PMC4511803; doi:10.1371/journal.pone.0132232)

**S3. Classification of ecosystem services along the rival/excludable gradient**

We observed that provisioning services (food production, raw materials, and freshwater) were rival and excludable services, because they were provided in particular (private) sites or given in concession to private operators. Food production and raw materials were excludable because they were obtained on private land and rival because their consumption made them less available for others. Freshwater supply was partially considered a non-rival and non-excludable service, but the fact that the amounts of water in a river are limited, made it a rival service. Moreover, as water intake is regulated by the regional water management body (a government office), its use can be allowed or restricted to certain petitioners by water concessions, becoming an excludable service.

Supporting ecosystem properties and most regulating services were non-excludable, as they have public access. However, these can be congestible given the limited capacity of ecosystems to buffer their overuse; for instance, streams have a limited capacity for maintaining good water quality after their use. Soil conditions, nutrient regulation, and habitat quality were partially included in this category, because by providing rights to access the land, these services are free to use (non-excludable); however, land property rights can exclude some stakeholders from them. In addition, high levels of use can deplete soil good conditions and the capacity to regulate nutrient cycling, causing a decrease in habitat quality (i.e., becoming a congestible service). Exceptions were biological control, which was considered non-rival because it cannot be consumed, and carbon sequestration, which at the small spatial scale of this study is not congestible.

Cultural ecosystem services (aesthetics, recreation, and environmental education) were partially non-rival (i.e., can benefit many people at the same time without being consumed) and non-excludable, as enjoying the landscape is usually open access. However, these can also be excludable, as some specific sites in our study area required an entry ticket or permit (e.g., fishing permits, entry to Monasterio de Piedra), and rival when available in a limited space or if an excessive number of users decrease their value.

**Figure S3. Ecosystem services classified as excludable/rival and related to each stakeholder group.** Colors indicate the type of ecosystem services (green=regulating, gold=provisioning, purple=cultural) and supporting ecosystem properties (blue). Intermediate regulating services are dashed and final services are solid. Rival and excludable services are in rectangles, non-rival and non-excludable services are in ellipses, and congestible services (non-excludable that can move from non-rival to rival) are in parallelograms. Impaired ecosystem services are in red, ecosystem services managed or co-produced are in bold, and they are marked with an asterisk (*) when managed by a single group. Note that habitat quality and carbon sequestration were only indirectly used by groups 1, 2 and 3, and that all ecosystem services linked to group 4 (excluding environmental education) were used indirectly.


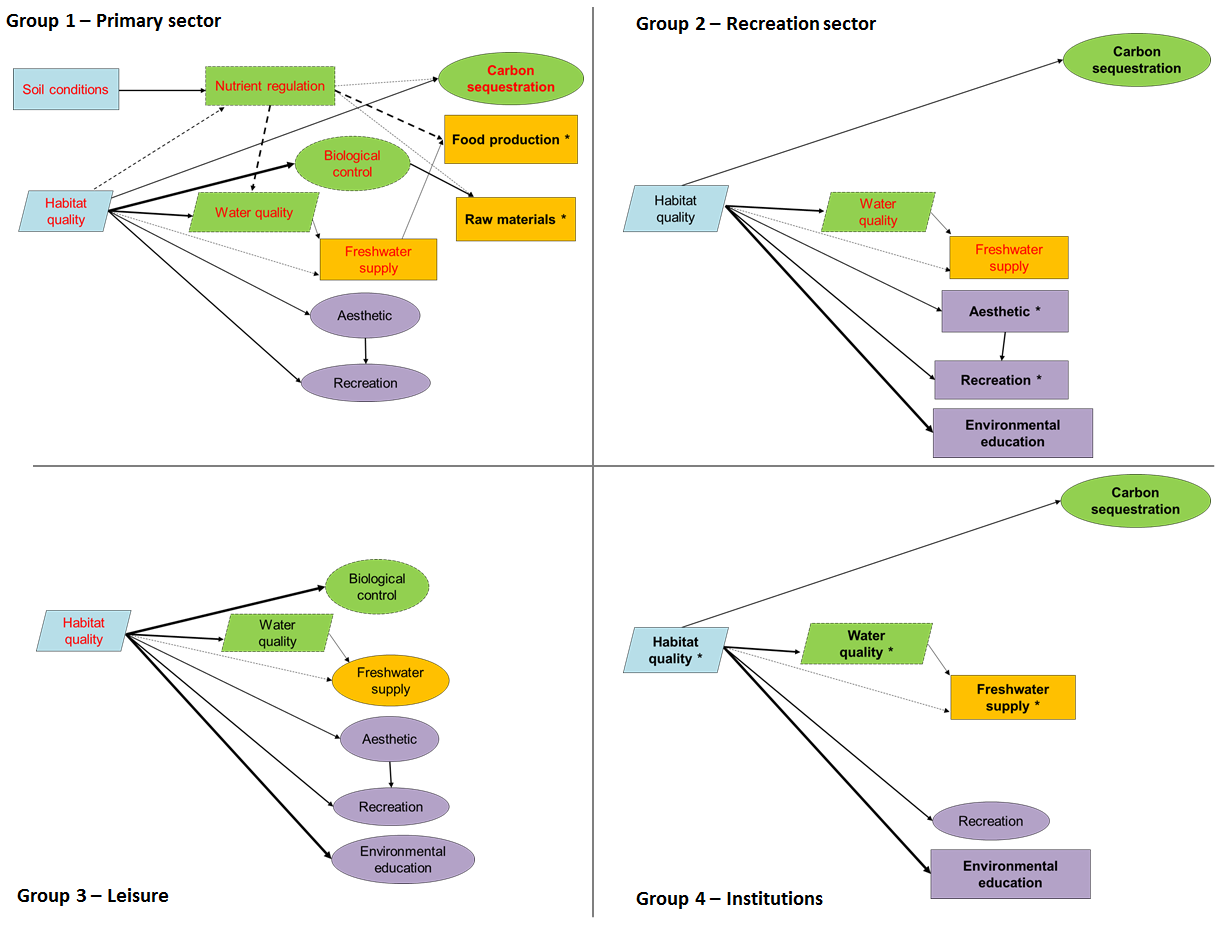

Supplement: S3 File — (DOCX) [file pone.0132232.s003.docx]
